# Lipase as a Chiral Selector Immobilised on Carboxylated Single-Walled Carbon Nanotubes and Encapsulated in the Organic Polymer Monolithic Capillary for Nano-High Performance Liquid Chromatography Enantioseparation of Racemic Pharmaceuticals

**DOI:** 10.3390/molecules28186663

**Published:** 2023-09-16

**Authors:** Ali Fouad, Frady G. Adly, Moustafa K. Soltan, Ashraf Ghanem

**Affiliations:** 1 Pharmaceutical Chemistry Department, Faculty of Pharmacy, Al-Azhar University, Assiut 71524, Egypt; alifouad247@gmail.com; 2 Complementary & Over the Counter Medicines Branch, Medicines Regulation Division, Therapeutic Goods Administration, Australian Department of Health and Aged Care, Canberra, ACT 2609, Australia; frady.gouany@health.gov.au; 3 Department of Medicinal Chemistry, Faculty of Pharmacy, Zagazig University, Zagazig 44519, Egypt; mostafakhames3@yahoo.com; 4 Oman College of Health Sciences, Ministry of Health, Muscat 132, Oman; 5 Chirality Program, Faculty of Science and Technology, University of Canberra, Canberra, ACT 2601, Australia

**Keywords:** lipase, carbon nanotubes, monolith, enantioseparation, encapsulation, immobilisation, nano-HPLC, resolution

## Abstract

Herein, we report the preparation of lipase immobilised on single-walled carbon nanotubes (SWCNTs) as an enantioselector for capillary monolithic columns and their application in the chiral separation of racemic pharmaceuticals. The columns were prepared through the encapsulation of functionalised SWCNTs (c-SWCNTs) within an organic monolithic polymer, followed by the immobilisation of lipase over the obtained monolith, over a three-day (L1) and five-day (L2) period. The prepared columns were tested for the enantioselective nano-HPLC separation of 50 racemic drugs. A suitable resolution was achieved for 25 drugs using nano-RP-HPLC conditions for both the L1 and L2 capillaries, while no specific resolution was detected under normal-phase HPLC conditions. The developed c-SWCNT-lipase-based polymeric monolithic capillaries are a promising expansion for separating pharmaceutical enantiomers’ using nano-HPLC.

## 1. Introduction

Drug enantiomers express different binding interactions with biological receptors and might exhibit a considerable number of other pharmacokinetic and pharmacodynamic properties. As such, regulatory authorities, such as the US Food and Drug Administration (FDA) [1], the European Medicines Agency (EMA) [2], and the Australian Therapeutic Goods Administration (TGA) [3], require a complete pharmacological and toxicological assessment of the individual drug enantiomers to assess the relevance of stereoisomerism for its effects and fate in vivo. This is regardless of whether the drug substance will be marketed as a pure enantiomer or as a racemate. These enforcements from regulatory authorities provoked the development of several robust, reliable, environmentally benign, and economically feasible separation techniques to analyse chiral pharmaceutical drugs [4,5,6,7].

In that respect, nano-HPLC has emerged as a microfluidic separation technique that involves a reduction in both column inner diameter (capillaries of 10–300 μm ID) and flow rate (200–1000 nL/min), sometimes reducing total analysis time, and reagents consumption, and being more suitable for the analysis of small samples [8,9]. Furthermore, the introduction of monolithic stationary phases in nano-HPLC [10,11,12,13] has offered superior advantages compared to conventional beaded supports, which include the preparation of capillary columns through in situ polymerisation with no frits required and, hence, avoiding frits-associated problems such as permeability, fragility, non-specific interactions, and manufacturing problems [14]. Although two of the main types of monolithic capillary columns are available, namely polymer-based and silica-based monolithic columns [15], a comparison between the technically challenging procedures for silica-monolith preparation and the simple and rapid process of preparing polymer-based monoliths with their high permeability and selectivity renders them an attractive support in the field of separation science [16,17,18]. 

To introduce chirality to polymer-based monolithic supports, a chiral selector (CS) is either copolymerised as a functional monomer [19] or adsorbed, immobilised, or coated on the monolithic surface after its preparation [20]. In that respect, several categories of CSs were reported for the enantioselective separation of racemates, for example, molecularly imprinted polymers, ligand exchange, brush-type, macrocyclic antibiotics-based, protein/glycoprotein-based, cellulose/amylose derivatives, and cyclodextrin(s) derivatives [7]. Our research group has also contributed to the field by reporting the use of carbamylated cyclodextrins [21], amylose [22], and macrocyclic antibiotics, including colistin sulphate, daptomycin, and polymyxin B [23,24,25,26], in polymer-based monolithic capillary columns, with their successful application in the nano-HPLC separations of multiple important pharmaceutical racemates.

In this article, we are continuing our contribution to the field by reporting the preparation of homogenous polymer monolithic supports containing *Candida antarctica* lipase B (CALB) immobilised on carboxylated single-walled carbon nanotubes (c-SWCNTs) for chiral nano-HPLC applications. 

Carbon nanotubes (CNTs) are allotropes of carbon, usually called graphite sheets, mainly consisting of sp^2^-hybridised carbon atoms. They are wrapped into cylindrical structures and commonly capped by a fullerene-like design. Once this cylinder is rolled into a single wall, it leads to single-walled carbon nanotubes (SWCNTs). However, multi-walled carbon nanotubes (MWCNTs) are formed from more than one wall [27]. Both types of CNTs (i.e., SWCNTs and MWNTs) were successfully used as stationary phases in Gas Chromatography [28,29], Liquid Chromatography [30], Capillary Electrochromatography (CEC) [31,32,33,34], and pseudo-stationary phases in Capillary Electrophoresis [34,35,36,37]. They have also been reported to have applications as sorbents in solid-phase and micro-extraction [38]. By adding specific features of the CNTs into the monolithic column, novel stationary phases with enhanced performances were obtained. Pre-treated SWCNTs were incorporated into vinyl-benzyl chloride (vbc)-based monolithic SPs for micro-LC and CEC [39]. MWCNTs have also been put into polymerisation mixtures with GMA and BMA as monomers [40,41]. 

Lipases have also been previously used for the chiral separation of enantiomers [42,43,44,45]. Being chiral in nature, lipases can interact with chiral analytes to form solids, with specific interactions leading to the successful resolution of enantiomers [46]. Two approaches were reported for the preparation of new polymer-based monolithic columns containing lipase as a chiral selector. They are either based on lipase immobilisation on the polymer-based monolith surface after preparation or the in situ encapsulation of lipase into the monolithic support during polymerisation [47].

Due to its high selectivity and broadened industrial applications, *Candida antarctica* lipase B enzyme (CALB) is widely used. Chiral columns based on *Candida antarctica* lipase B (CALB) were previously prepared by immobilising the enzyme on macro-porous silica gel [43,44,45] and on azlactone photo-grafted polymer monolith for biodiesel production [48].

## 2. Results and Discussion

### 2.1. Preparation and Characterisation of Functionalised Monolithic Capillaries

#### 2.1.1. Polymer Monolith Backbone: Preparation

**Figure 1:**
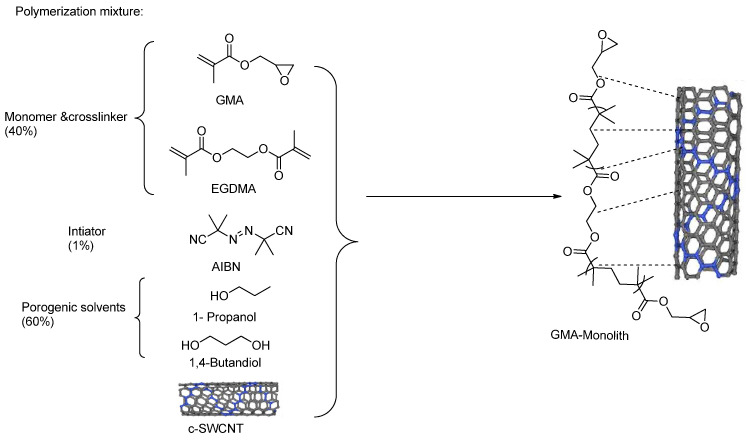
Schematic diagram show lipase immobilisation on the prepared CNT/GMA/EDMA-based monoliths.

Polymer monoliths were prepared via the in situ copolymerisation of GMA as an afunctional monomer and EDMA as a cross-linker in the presence of a ternary porogenic system composed of 48% 1-propanol containing c-SWCNTs (suspended at 100 μg/mL) and 12% 1,4-butanediol (Figure 1). The amount of monomers and the porogen were fixed at a ratio of 40:60, respectively, resulting in the obtained monoliths gaining a good balance of permeability, mechanical stability, and surface area [47,49,50]. The in situ polymerisation of the monolith in the fused silica capillary resulted in the trapping of c-SWCNTs within the produced monolithic structure. After monolith preparation, the epoxy monolithic capillaries were injected with a lipase solution (250 mmol/L) in a potassium phosphate buffer with a pH of 7.2 (10 mg/mL) at 0.25 μL/min for 72 and 120 h to produce the L1 and L2 capillaries, respectively.

#### 2.1.2. Characterisation of Functionalised Monolithic Capillaries

##### Microscopy

The prepared monolithic capillaries, L1 and L2, were investigated with light microscopy, demonstrating the homogeneous structure of the polymer inside the capillary.

**Figure 2:**
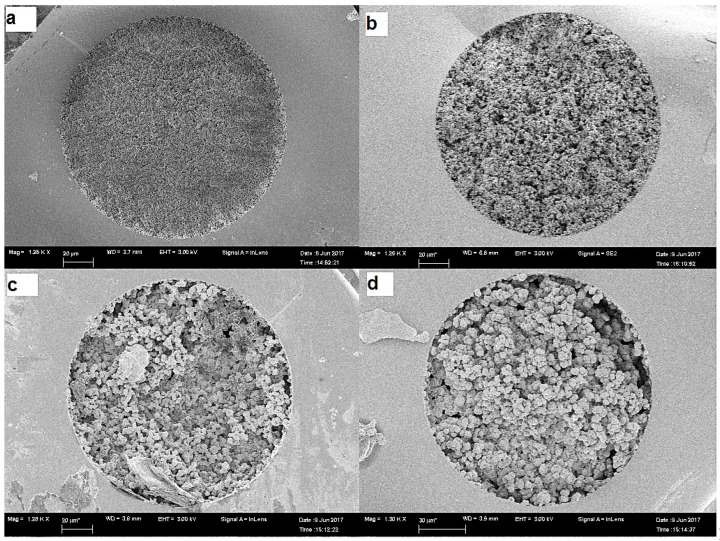
Scanning electron micrograph of (**a**) plain capillary, (**b**) CNT capillary, (**c**) L1 capillary and (**d**) L2 capillary at 25,000× showing different morphological properties.

**Figure 3:**
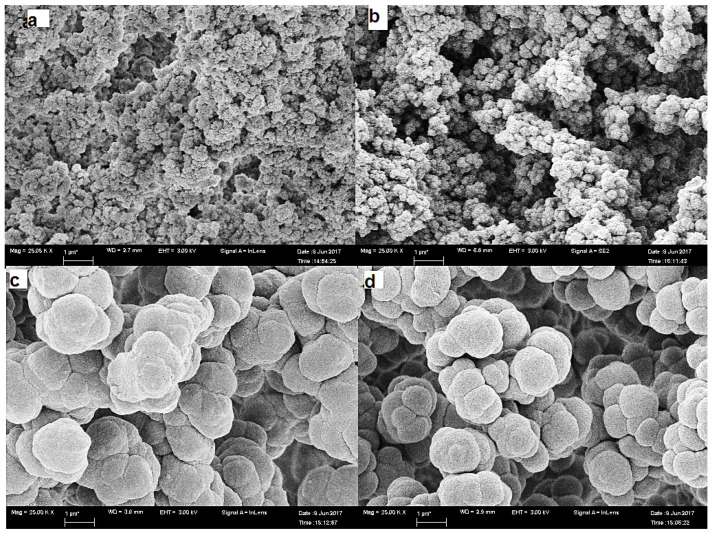
Scanning electron micrograph of (**a**) plain capillary, (**b**) CNT capillary, (**c**) L1 capillary, and (**d**) L2 capillary at 1200× showing different morphologic cal properties.

Scanning electron microscopy (SEM) has a vital role in studying the effect of the CS on the inner structure of the prepared monolithic capillaries. All prepared capillaries have inter-particle spaces in the uniform structures of the monolithic bed, with a heterogeneous surface of circular units inter-dispersed by large channels characteristic of the porous monolithic structures. Compared with plain and c-SWCNT-encapsulated monolithic capillaries (Figure 2a,b), the L1 and L2 capillaries have been shown to have relatively denser monolithic structures and soft microglobular surfaces caused by lipase coverage. Figure 2c,d and Figure 3c,d demonstrate the significant changes in the morphological properties caused by the immobilisation of lipase in the L1 and L2 capillaries.

##### Total Porosity

A partial characterisation of the prepared monolith quality through determining its total porosity (ƐT) was also carried out using the capillary HPLC flow method, where the unretained marker (uracil) was resolved using methanol as a mobile phase. 

The measured ƐT values for the columns L1 and L2 were 26.7% ± 1.9 and 28.4% ± 2.09, respectively.

The ƐT column porosity for the L1 and L2 monoliths was acceptable. Since the percentage of porogenic solvents in the polymerisation mixture was constant, this is attributed to using c-SWCNTs and lipase with a high-molecular-weight functional monomer. Additionally, it is well established that using a high chiral selector concentration gave rise to a capillary with reduced efficiency [51]. The optimum concentration of lipase and c-SWCNTs in the polymerisation mixture was 10 mg/mL and 100 µg/mL, respectively. The results revealed that a concentration of 10 mg/mL obtained a better resolution, while increasing the concentration to 40 mg/mL or more resulted in poor capillary efficiency; actually, the capillary was blocked due to the agglomeration or deposition of lipase and/or c-SWCNTs inside the capillary, so the washing step by methanol was not completed. The measured ƐT values for the columns L1 and L2 are listed in Table 1.

The nitrogen content in the L1 and L2 polymers was also determined through elemental analysis. This measurement gives a rough conclusion about the content of the capillary protein (i.e., lipase). The elemental analysis for the L1 and L2 polymers resulted in nitrogen contents of 2.25% and 2.41% (*w*/*w*), respectively. These results are higher than the nitrogen content observed for a blank capillary (i.e., 1.8% (*w*/*w*)). This increase in nitrogen content can be solely attributed to the immobilization of lipase on the monolith’s surface. Furthermore, the obtained nitrogen contents indicate that the protein content immobilized on the surface of the L2 polymer is higher than that of the L1 polymer, which is expected as the L2 polymer is known to have a higher content of lipase immobilized on its surface. The corresponding nitrogen content is listed in Table 2.

##### Mechanical Stability

**Figure 4:**
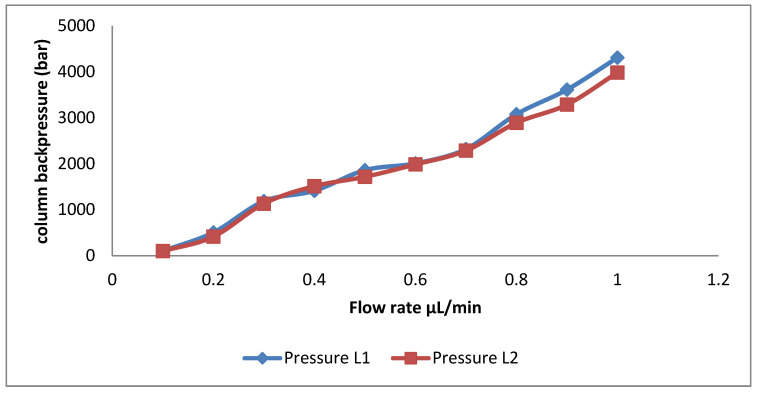
Column backpressure vs. mobile phase flow rate for L1 and L2 columns.

The mechanical stability of the prepared capillaries was tested using a mixture of methanol/water (80:20 *v*/*v*) with a flow rate of 0.1–1 µL/min. The function of the flow rate of the mobile phase was presented as a semi-linear increase in backpressure. Therefore, the high-pressure values did not cause any collapse in the packing monolith. The relation between the backpressure (values up to 5000 bar) versus the flow rate (from 0.1 µL/min to 1 µL/min) of a mixture of methanol/water is shown in Figure 4. The tested capillaries obtain a high mechanical stability over the pressure ranges (Figure 4).

### 2.2. Enantioseparation of Different Classes of Pharmaceutical Racemates under Multimodal Elution

The prepared L1 and L2 capillaries were tested in the chiral nano-HPLC resolution of 50 racemic drugs of different classes, including drugs acting on the autonomic nervous system and cardiovascular system, which include β- and α-blockers, dopamine antagonists, norepinephrine-dopamine reuptake inhibitors, antiarrhythmics, and catecholamines, as well as NSAIDs, antifungals, sedative-hypnotics, diuretics, antihistamines, anti-cancers, flavonoids, and other miscellaneous drugs. 

**Figure 5:**
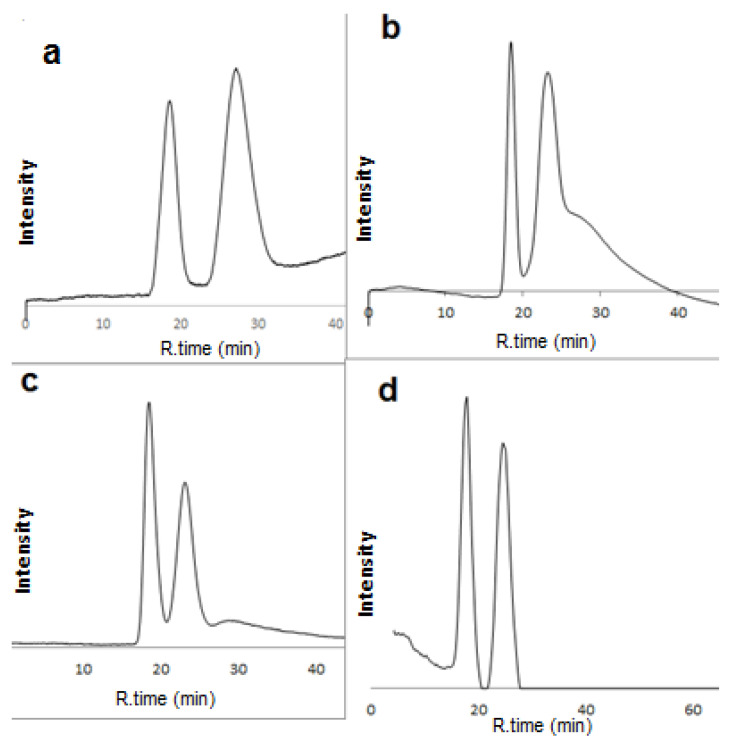
Enantioselective nano-LC separation of racemic hexaconazol (**a**) Mobile phase: methanol 100%, acebutolol (**b**) Mobile phase: methanol/water 80:20 *v*/*v*, glutamic acid (**c**), Mobile phase: methanol/water 30:70 *v*/*v* and diniconazol (**d**), Mobile phase: methanol /water 5:95 *v*/*v* on L1 capillary column (150 µm ID, 25 cm length). UV: 219 nm; flow rate: 1 µL/min.

C-SWCNTs and lipase were investigated to determine their performance as chiral selectors using the RP and NP chromatographic conditions. Firstly, the RP condition was checked using acetonitrile/water mixtures, but no significant chiral resolution was achieved. In addition, both acidic and basic additives were used without achieving any baseline separations. Furthermore, the mobile phase was composed of methanol 100% or methanol/water mixtures. Therefore, the mobile phase composed of methanol 100% or methanol/water mixtures at different concentrations varied from 5 to 95% (*v*/*v*). Twenty-four drugs were separated using the methanol-based mobile phase with acceptable resolutions (Rs ≥ 1) at different methanol percentages. A total of 14 of these 24 enantiomers gave baseline resolutions, including alprernolol, 1, atenolol, 3, acebutolol, 6, ibuprofen, 23, hexaconazol, 26, miconazole, 27, diniconazole, 28, ifosfamide, 29, normetanephrine, 30, nomifensine, 34, phenylalanine, 36, glutamic acid, 37, flavanone, 39, o-methoxy mandelic acid, 43, 4-hydroxy mandelic acid, 44, and clopidogril, 50 (Figure 5). It was observed that the separation efficiency in terms of separation factor and resolution for column L2 was generally better than for column L1. The enantioselective nano-LC separations of eighteen drugs are shown in Supplementary Figures S1, S2 and S4. The blank capillary did not show any enantioselective separation.

Otherwise, the NP consisting of *n*-hexane and isopropanol mixtures (90/10–60/40, respectively) with and without the addition of 0.1% TFA was used to resolve the tested racemates. No acceptable resolution was observed for any of the investigated racemates. These results focused on the polarity of the solvent in providing the molecular environment suitable for chiral discrimination. The separation (α) and resolution (Rs) factors for the baseline separated drugs and their resolution chromatographic conditions are listed in Table 3. The separation (α) and resolution (Rs) factors for the baseline separated drugs are calculated according to the following Equations (1) and (2).

(1) $$\alpha =\frac{k_{2}}{k_{1}}$$

where k_2_ is the retention factor of the second peak, and k_1_ is the retention factor for the first peak

(2) $$R=2\;\frac{t_{R2}-t_{R1}}{w_{1}+w_{2}}$$

where t_R_ is the peak retention time, and w is the peak width

**Figure 6:**
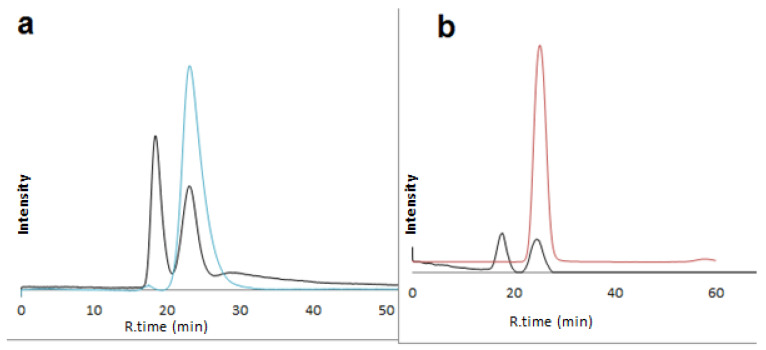
Enantioselective nano-LC separation of racemic diniconazol (**a**) on L1 capillary (black) and blank capillary (blue) using mobile phase: methanol/water 5:95 *v*/*v*, and glutamic acid (**b**) on L1 capillary (black) and blank capillary (orange) using mobile phase: methanol/water 30:70 *v*/*v*. UV: 219 nm; flow rate: 1 µL/min.

Although both lipase and c-SWCNTs were used previously as chiral selectors, confirmatory tests were carried out to exclude the non-presence of a chiral selector in situ in the prepared capillary monolith. The enantioselective separation was not achievable on the plain monoliths. The injected drugs on the plain column gave a single peak under the same chromatographic conditions (Figure 6). Furthermore, this confirms the absence of separated impurities or faulty separation due to some differences in peak areas of some resolute racemates, as shown in Supplementary Figure S3.

Lipase and c-SWCNTs were previously used as CSPs for the separation of different classes of racemic pharmaceuticals, such as normetanphrine, nomifensine, miconazole, and 4-hydroxy mandelic acid, using nano-HPLC [47] and were also used to resolve some chiral compounds in CEC [52,53]. The functionalisation of SWCNTs resulted in an increased number of carboxylate groups (COOH), which is vital in enantioseparation. This is confirmed by improved enantioseparation regarding separation factor and resolution and the broader spectrum of resolved racemates. It is well established that the use of lipase and c-SWCNTs in monolithic capillaries has the unique advantages of monoliths, the characteristics of lipase, and the properties of c-SWCNTs, as well as the environmentally benign manner of capillaries with negligible waste.

**Figure 7:**
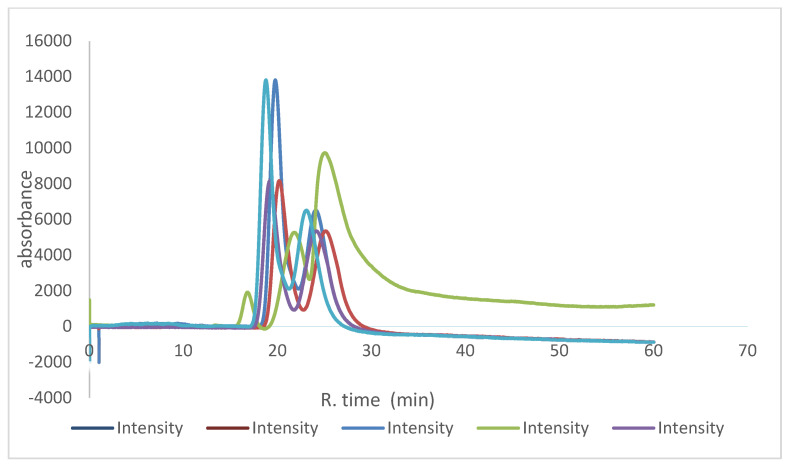
The loadability of the monolithic L2 capillary of o-methoxy mandelic acid started at run No. 10 and went up to run No. 109; mobile phase: methanol/water 80:20 *v*/*v*; UV: 219 nm; flow rate: 1 µL/min.

The repeatability of the prepared capillaries was investigated by preparing L2 capillaries under the same conditions to check the capillary-to-capillary repeatability and batch-to-batch repeatability. O-methoxy mandelic acid was selected to test the performance of the capillaries in terms of repeatability, as it had an acceptable resolution on both capillaries. The reproducibility of the retention times and resolution of both o-methoxy mandelic acid peaks were satisfactory. In the run-to-run repeatability, the average *Rt* for peaks one and two were 19 min (RSD = 0.6%) and 23.6 min (RSD = 0.4%), respectively, and the average *Rs* value was 1.35 (RSD = 1.5%). In capillary-to-capillary repeatability, the average Rts for peak one and peak two were 18.9 min (RSD = 1.5%) and 23.1 min (RSD = 0.6%), respectively, and the average Rs value was 1.33 (RSD = 3.3%). In batch-to-batch repeatability, the average retention times for peaks one and two were 19.4 min (RSD = 2.1%) and 23.6 min (RSD = 3.6%), respectively, and the average Rs value was 1.3 (RSD = 4.1%). These results suggest that the monolithic capillaries can be used for reproducible enantioseparation. In addition, the capillary was injected for more than 120 runs on the same column to ensure good loadability; o-methoxy mandelic acid was injected in different orders, starting at run number 10 and ending at run number 109. A similar separation was obtained (Figure 7).

### 2.3. Discussion

Nano-HPLC is considered a green analysis approach due to its small solvent consumption and reduced waste production compared to conventional HPLC. The function of the CS in achieving a good separation for the racemates confirms specific fundamentals for its chiral recognition mechanism [25,54]. In the present study, SWCNTs were carboxylated according to the reported procedure to improve their dispersion properties. The prepared c-SWCNT was inserted into the organic polymer support as the CS. The chiral recognition capabilities of the c-SWCNT as new CSPs were tested under different interaction modes between the analytes and the CSPs. 

The enantioselective capabilities of c-SWCNTs as new CSPs were investigated under diverse interaction modes between the analytes and the adsorbent. The enantioseparation results observed were much higher than those obtained with the employment of CNTs as a pseudo-stationary phase in CE to separate norephedrine isomers [27] and better than the results obtained using SWCNTs in nano-HPLC that were followed by our group [52,53]. This may be due to the increased number of carboxylate groups, which improves the enantioseparation as described previously [52,53]. Nanotubes have been used recently to improve the enantioseparation performance of chiral selectors such as chitosan [52,53]. This is attributed to its unique properties, such as increased surface area and the number of carboxylate groups that make ionic bonds with the other CSs. It also has a vital role in chiral recognition of the analytes.

Under the alkaline condition of the potassium phosphate buffer, the highly nucleophilic free amino groups of lipases will attack the monolith surface’s reactive epoxy functional groups, leading to lipase’s covalent bonding to the monolith surface. To confirm the complete lipase coverage for the monolithic surface groups, the lipase was pumped for 3 and 5 days. The unreacted epoxy groups played a negligible role in the chiral separation, which was confirmed by running the blank epoxy-based columns. In addition, the free amino groups of the lipase will attack the carboxylate groups of the c-SWCNTs.

The chiral recognition abilities of lipase could be due to the presence of ionic, H-bond donor/acceptor, and hydrophobic interactions within the enzyme active site, in addition to the size of the enzyme active site. 

**Figure 8:**
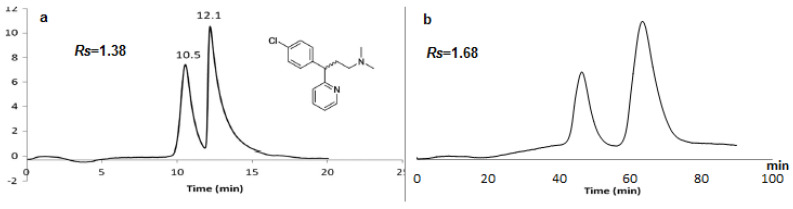
Enantioselective nano-LC separation of chlorpheniramine on a M1L capillary column (150 μm ID, 25 Cm length). Mobile phase: methanol/water (0.1% TFA) 30:70 *v*/*v* (**a**) (ref. [47]) and chlorpheniramine on L2 capillary column (150 μm ID, 25 Cm length). Mobile phase: methanol/water 20:80 *v*/*v*; (**b**) UV: 219 nm; flow rate: 1 µL/min.

By using the SWCNTs in the nano-HPLC performed by our group, only seven drugs achieved baseline separation; furthermore, with the single use of lipase in nano-HPLC previously reported by our group, twelve drugs obtained an acceptable separation of alprenolol, atenolol, carbuterol, chlorpheniramine, cizolertine, desmethylcizolertine, carbinol, 4-hydroxy-3-methoxymandelic acid, bromoglutithimide, nomifensine, normetanephrine, and sulconazole using RP-HPLC [47]. At the same time, the use of lipase and c-SWCNTs in monolithic capillaries has lipase characteristics, e.g., the large size of the enzyme active site, and the properties of c-SWCNT, e.g., the large surface area and the increased number of carboxylate groups. This resulted in the enantioseparation of twenty-four drugs; nineteen of which had a significant resolution (*Rs* ≥ 1), as shown in Table 3. A better separation was obtained by the L2 capillary and a few by the L1 capillary, so pumping lipase into the L2 capillary for five days is better to ensure adequate lipase coverage for the epoxy surface groups. By comparing the separation of chlorpheniramine obtained using lipase only (Figure 8a) [47] with that obtained using lipase in the presence of c-SWCNTs (Figure 8), the obtained result confirmed the superiority of using lipase in the presence of c-SWCNTs over using either lipase or SWCNTs separately. Furthermore, fluribiprofen and ifosfamide drugs were investigated using lipase-based capillaries, as in reference [47], without achieving any significant resolution, while when using lipase in the presence of c-SWCNTs (Figure 8), both of them obtained baseline separations in Supplementary Figure S2a,b in the Supplementary Materials. 

Investigating the prepared capillaries with a mixture of acetonitrile and water as the mobile phase in the 10–90% *v*/*v* range did not achieve any significant enantioselective resolutions. However, enantioselective separation was observed when a methanol-based mobile phase was used. This confirms the importance of solvent polarity in the chiral recognition mechanism. Furthermore, the use of buffers in mobile phases had a negative effect on the capillaries’ lifetime and their potential problems with nano-LC systems. The enantioseparation results were observed with long analysis times; this is because the Shimadzu LC-10AD VP pump used did not load more than a 6000 bar back pressure. Therefore, it was not possible to use higher flow rates to avoid the backpressure exceeding its maximum value.

The presence of a shoulder after the second peak in the acebutolol separation in Figure 5b can be attributed to several factors. Since all separated racemates have the same sample concentration, injection volume, and optimized LC conditions, all these parameters were excluded. Here, we may attribute the presence of this shoulder to the presence of contamination in the sample or from the internal components of the capillary, and the analytical method needs to be optimized in further studies on the prepared CSP. 

The enantioseparation results observed were much higher than those obtained by employing c-SWCNT and lipase separately as CS phases in the nano HPLC carried out by our group [25,26,54]. It is well established that the use of lipase and c-SWCNTs in a monolithic capillary has the unique advantages of monoliths, including the characteristics of lipase and the properties of c-SWCNTs, as well as the environmentally benign manner of capillaries with negligible waste.

## 3. Experimental Section

### 3.1. HPLC Conditions

Water/methanol (*v*/*v*) and acetonitrile/water (*v*/*v*) mobile phases were used in RP-HPLC, while the *n*-hexane/2-propanol mobile phase was used in NP-HPLC. The injection volume used for all drugs was 1 µL, and UV detection was recorded at 219 nm wavelength. 

#### 3.1.1. Reagents

The chemicals and reagents used in this study were purchased from various suppliers. Ethylene glycol methacrylate (EDMA, 98%), 3-(trimethoxysilyl) propyl methacrylate (98%), 1-propanol (99%), 1,4-butanediol (99%), methacryloyl chloride (97%), trifluoroacetic acid (TFA, ≥99.5%), sodium hydroxide, and hydrochloric acid were purchased from Aldrich (Milwaukee, WI, USA). Novozym 525 L (activity 5000 U/g) was obtained from Novo Nordisk (Bagsvaerd, Denmark). Single-walled carbon nanotubes, with dominant (6,5) and (7,6) chiralities, were purchased from Sigma-Aldrich (Milwaukee, WI, USA). Acetone (AR grade) and ethanol (HPLC grade) were purchased from BDH (Kilsyth, Vic., Australia). Methanol (HPLC grade) was purchased from Scharlau (Sentmenat, Spain). 2,2-Azobis(isobutyronitrile) (AIBN) was obtained from Wako (Osaka, Japan). All water used for dilutions and experiments was purified using a Nano-pure Infinity water system (NJ, USA). Racemic analytes mainly were purchased from Sigma-Aldrich. The fused-silica capillaries with a 150 µm internal diameter were purchased from Polymicro Technologies (Phoenix, AZ, USA). 

#### 3.1.2. Instrumentation

The nano-LC system was a Shimadzu LC-10AD VP pump (Kyoto, Japan), and a Rheodyne injector model 7725i-049 (Park Court, CA, USA), a GL Science UV-Vis detector model MU 701 UV-VIS (Tokyo, Japan), and a Shimadzu CDM-20A communications bus module (Kyoto, Japan) was used. The system flow was split after direct injection. The Shimadzu Lab-Solutions software version 5.54 SP2 (Kyoto, Japan) processed the data. 

### 3.2. Preparation of the Monolithic Columns

#### 3.2.1. Preparation of the Carboxylated SWCNT (c-SWCNT)

The c-SWCNTs were prepared according to the reported procedure [22] with minor modifications. Briefly, 100 mg of SWCNTs were suspended in 20 mL of a 3:1 H_2_SO_4_:HNO_3_ mixture, ultrasonicated (50 W, 60 Hz) for 2 h, highly diluted with water (up to 2 L) and filtered through a 0.2 µm PTFE membrane. The filtered c-SWCNTs are washed with water, put in 50 mL of 1 M HCl solution, sonicated for 1 h, filtered, washed with water, and air dried. The obtained nanotubes were suspended in 2-propanol at 100 μg/mL and sonicated for 1 h immediately before addition to the polymerisation mixture.

#### 3.2.2. Activation of the Fused Silica Capillaries

The activation and surface functionalisation of the inner walls of the fused silica capillaries was carried out using our reported procedure [21,22,23,24,25,55]. In summary, the fused silica capillaries were cleaned and activated using a series of rinses with acetone, water, sodium hydroxide, and hydrochloride. They were then washed with water and ethanol, then a solution of 3-(trimethoxysilyl) propyl methacrylate was pumped through the capillaries. After washing with acetone and drying with nitrogen, the capillaries were left at room temperature for 24 h.

#### 3.2.3. Preparation of the c-SWCNT Functionalised Monolithic Columns

After the activation and surface functionalisation of the inner walls of the fused silica capillaries have been completed, the polymerisation mixture was prepared by mixing 20% Glycidyl methacrylate monomer (GMA), 20% Ethylene Glycol Dimethacrylate cross-linker (EDMA), and 60% porogens, which were divided into 48% 1-propanol containing c-SWCNT (suspended at 100 μg/mL) and 12% 1,4-butanediol (all percentages are *w*/*w*). To confirm a formal insertion and homogenous distribution of c-SWCNTs in the organic polymerisation mixture, a sonication process was carried out for c-SWCNT suspended in the porogenic solvents along with AIBN (1% *w*/*w*) before it was mixed with the GMA/EDMA monomer mixture. The polymerisation mixture was sonicated one more time for 1 h before it was injected into the capillary.

The ∼25 cm surface-activated capillaries were filled with the polymerisation mixture using a Harvard syringe pump at 250 nL/min. The polymerisation reaction took place at 70 °C in a water bath for 20 h, where the capillaries were tightly closed with a septum. When the polymerisation time had elapsed, excess reactants were flushed by pumping methanol through the capillaries at a flow rate of 100 µL/h for 72 h and then conditioned by pumping a mobile phase (methanol/water 30:70 *v*/*v*) for 24 h at 10 µL/h flow rate.

### 3.3. Immobilisation of Lipase on the Surface of the Carboxylated Single-Walled Carbon Nanotubes Organic Monolithic Support

Using the prepared GMA/EDMA-based monolithic capillaries, L1 and L2 capillaries were prepared by pumping a 10 mg/mL solution of lipase in potassium phosphate buffer (250 mmol/L; pH 7.2) through the monolithic capillaries at 0.25 μL/min for 3 and 5 days, respectively. Potassium phosphate buffer (250 mmol/L; pH 7.2) was pumped through the capillaries for 3 min to wash it and remove the unwanted particles, conditioned with the mobile phase (methanol/water 30:70 *v*/*v*) for 6 h, connected to the nano-HPLC system, and tested for enantioselective analysis.

The prepared monolithic capillaries, L1 and L2, were investigated with light microscopy to check the homogeneous structure of the polymer inside the capillary and confirm the absence of any air bubbles and unfilled parts inside the capillary.

#### 3.3.1. Scanning Electron Microscopy (SEM) and Total Porosity of the Prepared Monoliths

Scanning electron microscopy (SEM) was used to evaluate the impact of c-SWCNTs and lipase enzymes on the monolithic shape inside the prepared capillaries. The capillaries were divided into ~1 cm portions, placed perpendicularly on a 12.7 mm pin-type aluminium stub, and fixed using double-face epoxy resin tape. SEM high-resolution images were taken after coating the capillary samples with gold using SEM in the Centre for Advanced Microscopy, Australian National University.

#### 3.3.2. Partial Characterisation of the Prepared Monolith Quality through Determining Its Total Porosity (ƐT)

The capillary HPLC flow method was used to determine total porosity (ƐT), where the unretained marker (uracil) was run using methanol as a solution. The volume of the methanol was put in a closed vial to avoid errors due to its evaporation and weighed out in a given time at a 2 µL/min flow rate. The following equation determined the total porosity:

ƐT = V/(π∙r^2^ u) × 100

where ƐT is the total porosity, V (m^3^/s) is the methanol volume, r (m) is the inner radius of the empty capillary, and u (m/s) is the linear velocity of methanol as the mobile phase; it is obtained by dividing the effective length of the capillary by the retention time of unretained marker.

#### 3.3.3. Composition of Stock Solutions and Sample Solutions

Stock solutions of the racemic drugs tested were prepared in filtered HPLC grade methanol at a 1 mg/mL concentration. Before injection, the designed solutions were diluted ten times and filtered through Sartorius Minisart RC filters (0.2 µm pore size, Göttingen, Germany). The chemical structures of the 50 drugs tested are demonstrated in Supplementary Figure S5 [22,23].

## 4. Conclusions

Herein, c-SWCNTs were encapsulated into an organic polymer monolithic support, followed by immobilizing lipase over the organic polymer for 72 h (L1) and 120 h (L2). This can create a CSP with better chiral resolution than that obtained using either lipase or c-SWCNTs alone. The prepared lipase–c-SWCNT-based monolithic capillary has the unique advantages of monoliths, the characteristics of lipase, and the properties of c-SWCNTs, as well as the environmentally benign manner of capillaries with negligible waste. A stable c-SWCNT suspension was obtained in a solution through the carboxylation of SWCNT. Then, the in situ polymerization process was reached via thermal initiation. An immobilization procedure was carried out by covering the monolith with a lipase enzyme to obtain new affinity monoliths containing c-SWCNT and lipase as chiral selectors. The functionalised monolithic capillaries were investigated using light microscopy and SEM; their chromatographic performance was tested through separating the different pharmaceutical enantiomeric drugs.

Using the methanol-based mobile phase, twenty-four drugs were separated with acceptable resolution (Rs ≥ 1) at different methanol percentages. A total of 14 of these 24 enantiomers gave baseline resolutions, including alprernolol, 1, atenolol, 3, acebutolol, 6, ibuprofen, 23, hexaconazol, 26, miconazole, 27, diniconazole, 28, ifosfamide, 29, normetanephrine, 30, nomifensine, 34, phenylalanine, 36, glutamic acid, 37, flavanone, 39, o-methoxy mandelic acid, 43, 4-hydroxy mandelic acid, 44, and clopidogril, 50 (Figure 5). It was observed that the separation efficiency in terms of the separation factor and resolution for column L2 was generally better than for column L1. The blank capillary did not show any enantioselective separation. This mobile phase eliminates the use of harsh and expensive solvents. The newly prepared capillary columns as a part of nano-LC with reversed mobile phase produced a gentle separation technique in alignment with the concept of green chemistry. 

## Figures and Tables

**Table 1:** Porosity and performance of the prepared monolithic capillaries using uracil; mobile phase: methanol 100%, flow rate: 1 μL/min.

Column	ƐT %
L2	28.4 ± (2.09)
L1	26.7 ± (1.9)

**Table 2:** Measured nitrogen content in different columns.

Column	Nitrogen (% *w*/*w*)
G	1.8
L1	2.25
L2	2.41

**Table 3:** Chromatographic conditions and separation and resolution factors for the resolved racemates.

Phase	Capillary	Mobile Phase	Drug	*R_t_*1 (min)	*R_t_*2 (min)	Separation Factor (α)	Resolution (Rs)
Reversed phase	L2	Methanol:water 10:90	Miconazole	21	28	1.4	1.4
	L1		Aminoglutathemide			1.1	1.2
	L2		o-methoxy mandilic acid	23	37	1.3	1.9
	L1	Methanol:water 5:95	Flavanone	17	23	1.4	1.6
	L2	Methanol:water 20:80%,	Clopidogril	16	22	1.4	1.6
	L1		propafenone	24	30	1.2	1.2
	L2		Nomifensine	17	22	1.2	1.5
	L2		Chlorphinarmine	46	62	1.4	1.7
	L2	Methanol:water 80:20%,	Ifosfamid	18	23	1.3	2
	L1		Ampicilline	18	24	1.3	1.3
	L2		phenylalanine	19	25	1.3	1.8
	L1		Arternol	18	23	1.3	1.4
	L2		hexaconazole	18	27	1.4	1.6
	L1		Tocainide	19	23	1.3	1
	L2		diniconazole	18	24	1.4	1.8
	L1		Propranolol	19	24	1.3	1.3
	L2		Normatenphrine	18	24	1.4	1.9
	L2		Alprenolol	19	24	1.3	1.6
	L1		4-hydroxy mandlic acid	22	32	2	1.9
	L2	Methanol:water 50:50%	Ibuprofen	17	25	1.4	1.4
	L2		Glutamic acid	18	22	1.4	1.6
	L2		Atenolol	38	47	1.4	2.3
	L1	Methanol 100%	Fluribiprofen	16	23	1.2	1.3
	L1		Metoprolol	21	28	1.3	1.3

## Data Availability

The data presented in this study are available on request from the corresponding author.

## Supplementary Materials

The following supporting information can be downloaded at: https://www.mdpi.com/article/10.3390/molecules28186663/s1, Figure S1: Enantioselective nano-lc separation of racemic miconazol (a) (Mobile phase: Methanol:water 10:90 *v*/*v*, atenolol (b) (Mobile phase: methanol/water 50:50 *v*/*v*, Nomifensine (c), (Mobile phase: methanol/water 10:90 *v*/*v* and Normatenphrine (d), (Mobile phase: methanol/water 80:20 *v*/*v* on L2 capillary column (150 µm ID, 25 cm length). UV: 219 nm, flow rate: 1 µL/min.; Figure S2: Enantioselective nano-lc separation of racemic Fluribiprofen (a) (Mobile phase: Methanol:water 50:50 *v*/*v*), Ifosfamid (b), phenylalanine (c), and Propranolol (d), (Mobile phase: methanol/water 80:20 *v*/*v* on L2 capillary column (150 µm ID, 25 cm length). UV: 219 nm, flow rate: 1 µL/min.; Figure S3: Enantioselective nano-lc separation of racemic Nomifensine on L2 capillary (violet) and blank capillary (black) using mobile phase: methanol/water 20:80 *v*/*v*, UV: 219 nm, flow rate: 1 µL/min.; Figure S4: Enantioselective nano-lc separation of racemic tocainide (a), 4-hydroxy mandelic acid (b), clopidogrel (c), flavanone (d), alprenolol (e), propafenone (f), ibuprofen (g), ampicilline (h), metoprolol (i) and arternol (j) (Mobile phase: Methanol:water mixture *v*/*v* on L1 and L2 capillaries (150 µm ID, 25 cm length). UV: 219 nm, flow rate: 1 µL/min. Figure S5: Chemical structures of the tested racemic compounds
