# Supplementary material for: Lipase as a Chiral Selector Immobilised on Carboxylated Single-Walled Carbon Nanotubes and Encapsulated in the Organic Polymer Monolithic Capillary for Nano-High Performance Liquid Chromatography Enantioseparation of Racemic Pharmaceuticals
Source: Molecules. 2023 Sep 16;28(18):6663. doi: 10.3390/molecules28186663 (PMC10534468; doi:10.3390/molecules28186663)
Supplement: Supplementary file 1 [file molecules-28-06663-s001.zip › molecules-2587506-supplementary.pdf]

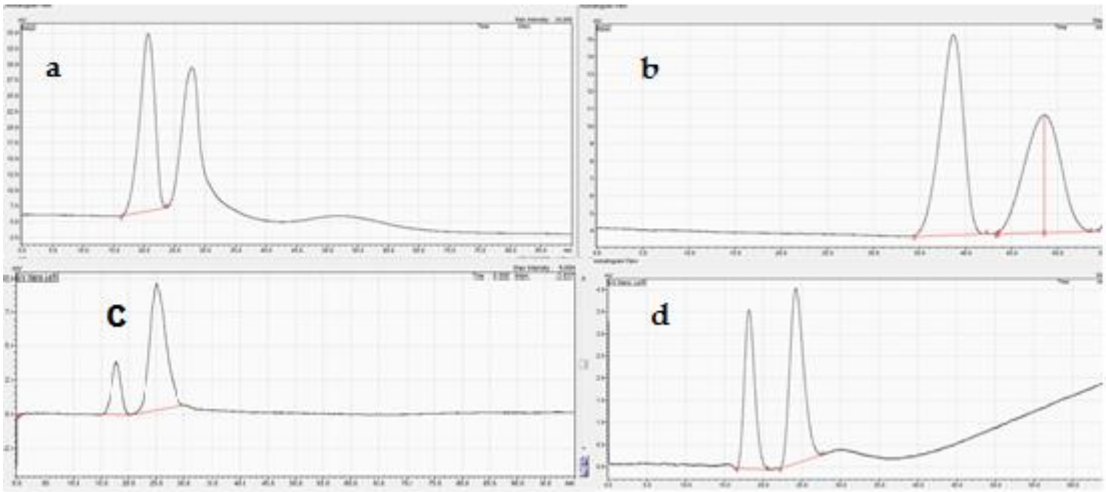

Figure 1: Enantioselective nano-lc separation of racemic miconazol (a) (Mobile phase: Methanol :water 10:90 v/v, atenolol (b) (Mobile phase: methanol /water 50:50 v/v, Nomifensine (c), (Mobile phase: methanol /water 10:90 v/v and Normatenphrine (d), (Mobile phase: methanol /water 80:20 v/v on L2 capillary column (150 µm ID, 25 cm length). UV: 219 nm, flow rate: 1 µL/min.

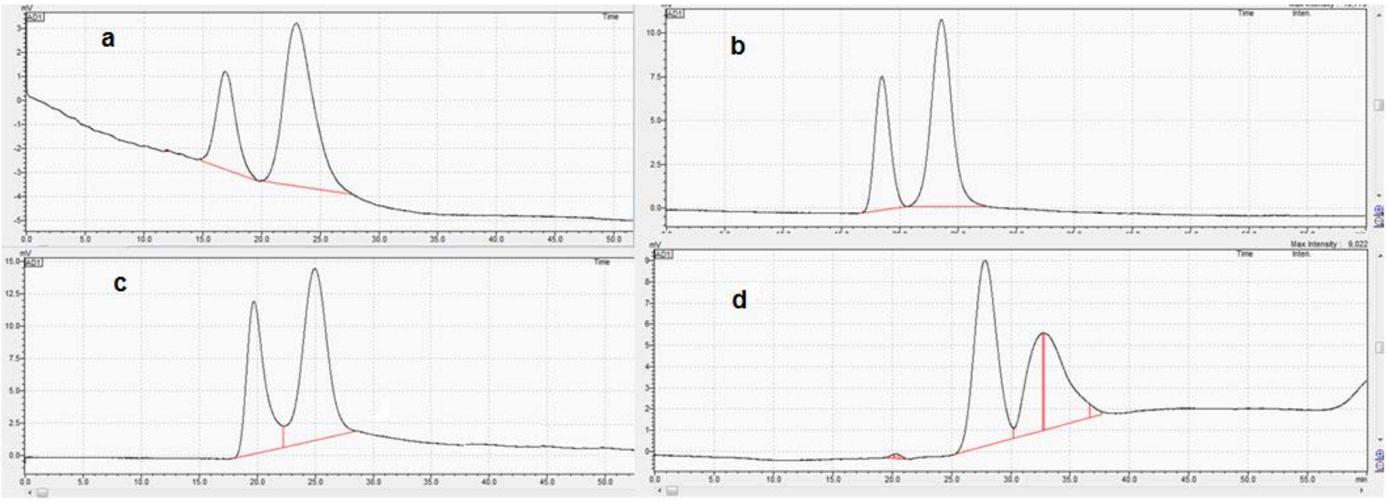

Figure 2: Enantioselective nano-lc separation of racemic Fluribiprofen (a) (Mobile phase: Methanol :water 50:50 v/v), Ifosfamid (b), phenylalanine (c), and Propranolol (d), (Mobile phase: methanol /water 80:20 v/v on L2 capillary column (150 µm ID, 25 cm length). UV: 219 nm, flow rate: 1 µL/min.

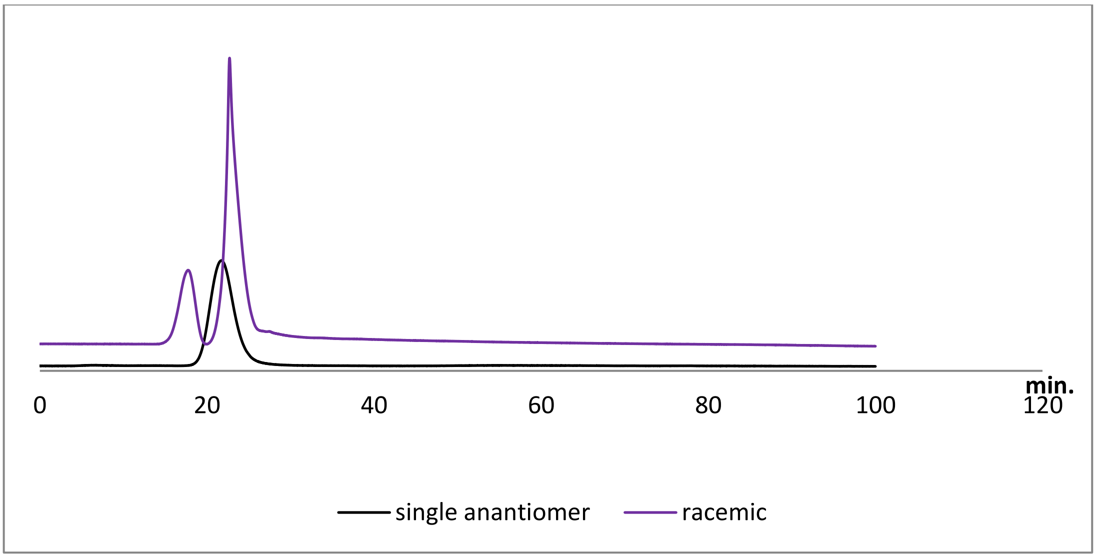

Figure 3. Enantioselective nano-lc separation of racemic Nomifensine on L2 capillary (violet) and blank capillary(black) using mobile phase: methanol /water 20:80 v/v, UV: 219 nm, flow rate: 1 µL/min.

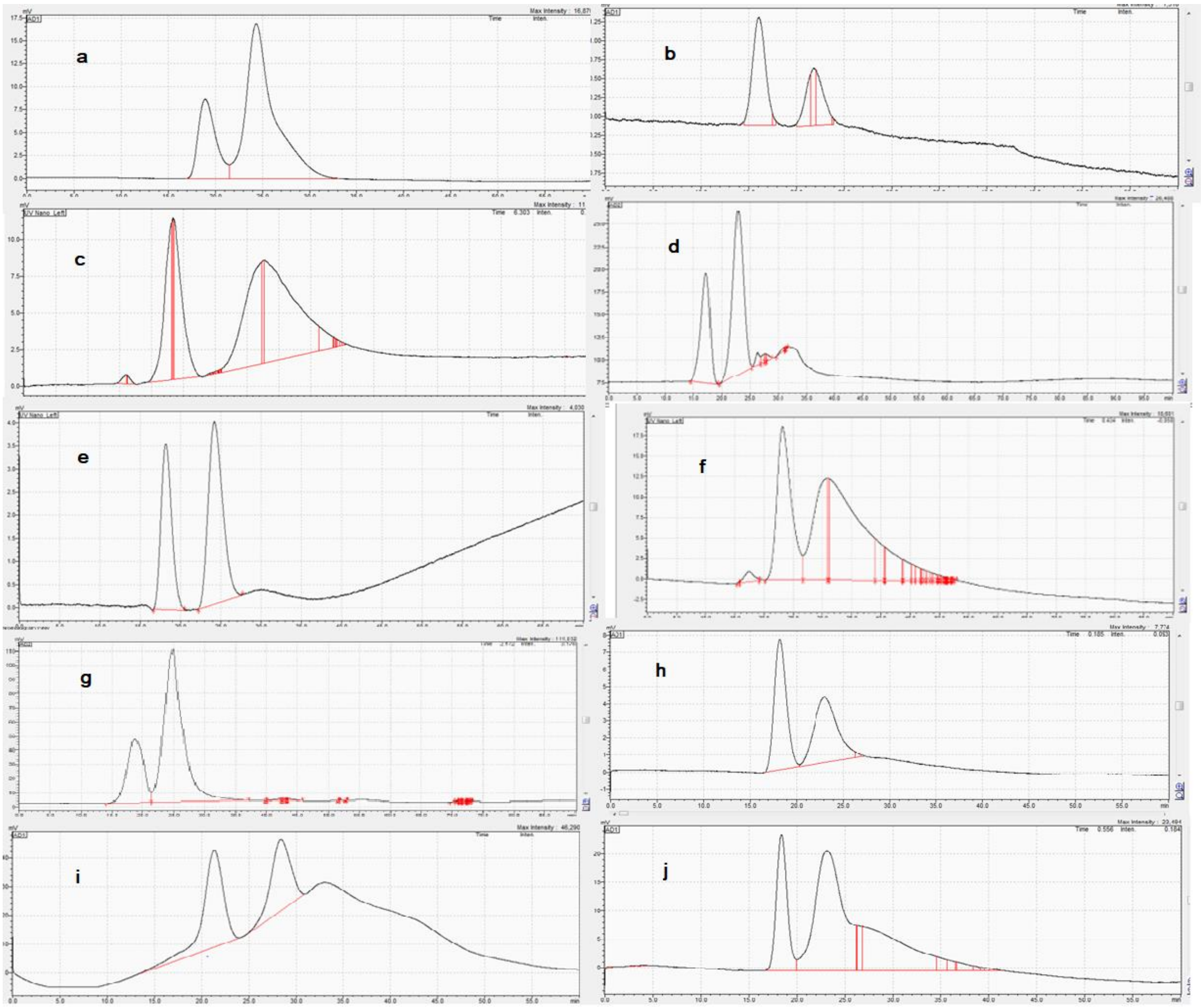

Figure 4: Enantioselective nano-lc separation of racemic tocainide (a), 4- hydroxy mandelic acid (b), clopidogrel (c), flavanone (d), alprenolol (e), propafenone (f), ibuprofen (g), ampicilline(h), metoprolol (i) and arternol (j) (Mobile phase: Methanol :water mixture v/v on L1 and L2 capillaries (150 µm ID, 25 cm length). UV: 219 nm, flow rate: 1 µL/min.

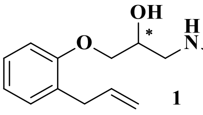 1 Alprenolol	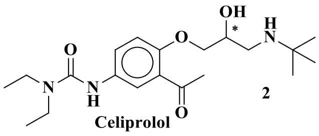 2 Celiprolol	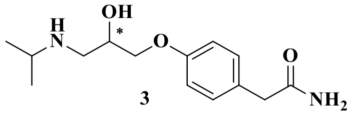 3 Atenolol	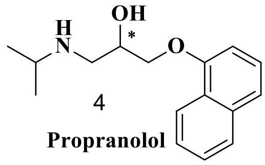 4 Propranolol
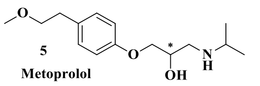 5 Metoprolol	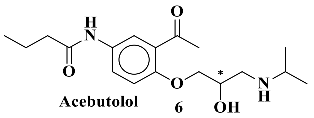 Acebutolol 6	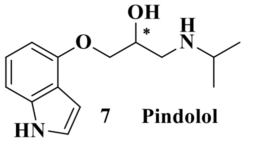 7 Pindolol	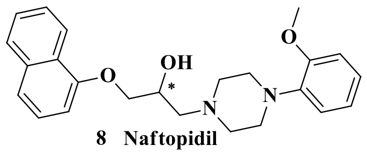 8 Naftopidil
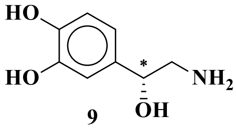 9 Norepinephrine	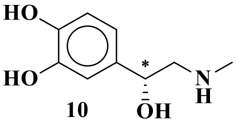 10 Epinephrine HCl	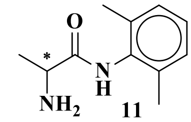 11 Tocainide	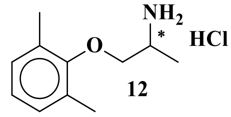 12 Mexiletine HCl
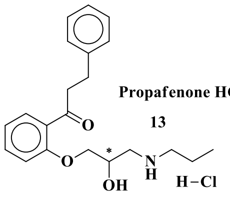 Propafenone HCl 13	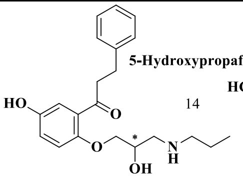 5-Hydroxypropafenone HCl 14	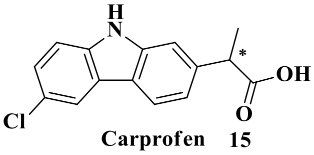 Carprofen 15	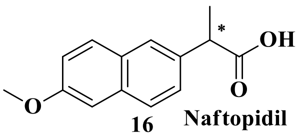 16 Naftopidil
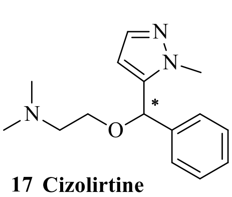 17 Cizolirtine	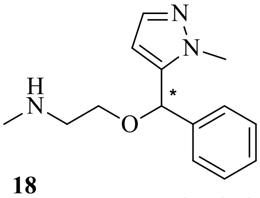 18 Desmethyl cizolirtine	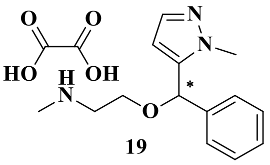 19 Cizolirtine oxalate	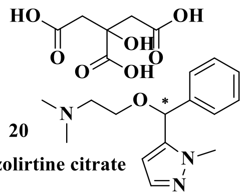 20 Cizolirtine citrate
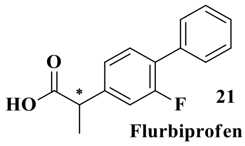 21 Flurbiprofen	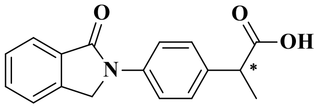 22 Indoprofen	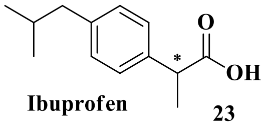 Ibuprofen 23	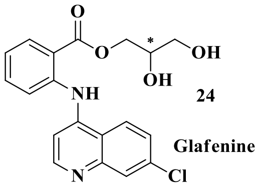 24 Glafenine
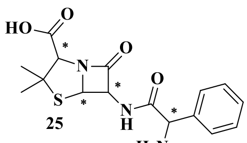 25 Ampicillin	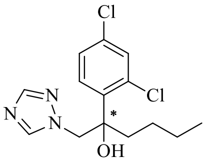 26 Hexaconazole	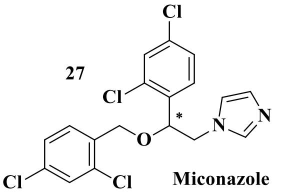 27 Miconazole	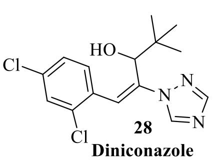 28 Diniconazole
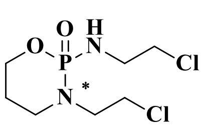 Ifosfamide 29	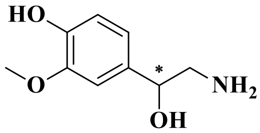 30 Normetanephrine	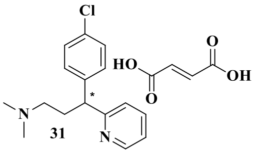 31 Chlorpheniramine maleate	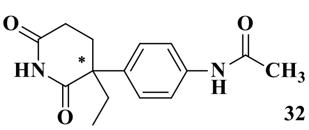 32 N-acetyl aminoglutethimide
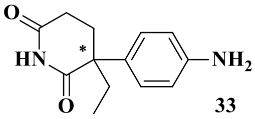 33 Aminoglutethimide	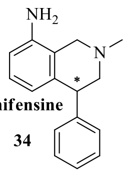 Nomifensine 34	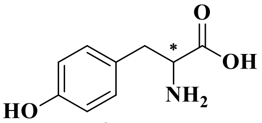 35 Tyrosine	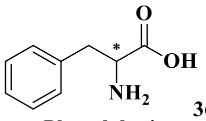 Phenylalanine 36
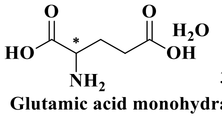 37 Glutamic acid monohydrate	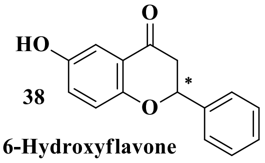 38 6-Hydroxyflavone	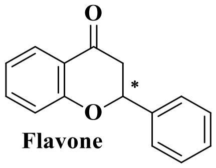 39 Flavone	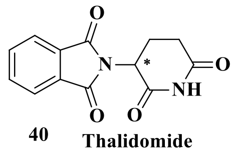 40 Thalidomide

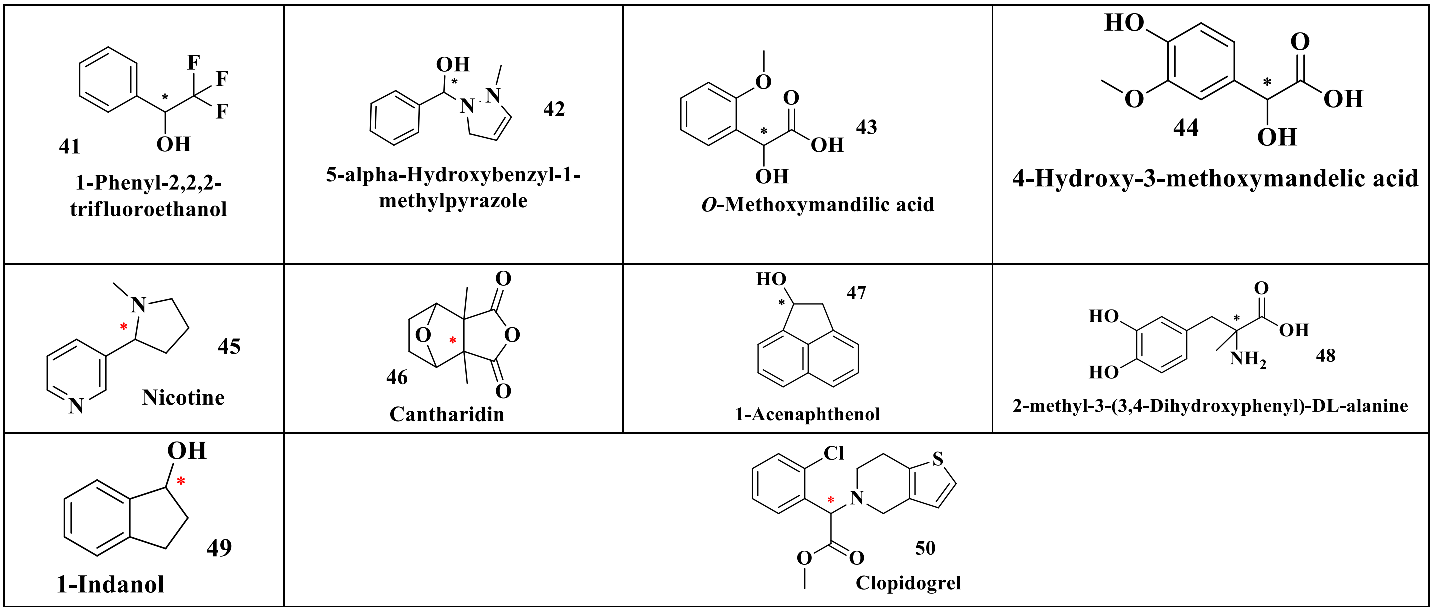

Figure 5: Chemical structures of the tested racemic compounds
